# Azole resistance in *Aspergillus* isolates from animals or their direct environment (2013–2023): a systematic review

**DOI:** 10.3389/fvets.2025.1507997

**Published:** 2025-03-20

**Authors:** Lucía Dieste-Pérez, Manon M. C. Holstege, Judith E. de Jong, Annet E. Heuvelink

**Affiliations:** Royal GD, Deventer, Netherlands

**Keywords:** *Aspergillus fumigatus*, *Aspergillus flavus*, azole resistance, animals, fungi

## Abstract

The resistance of *Aspergillus* species to azoles in human medicine is gaining increasing attention, and the role of animals and agricultural practices in this issue is becoming a significant source of concern. To gain better insights into the occurrence of azole resistance in *Aspergillus* spp. isolates from animals, a systematic literature review was conducted. Searches were conducted in the PubMed and Scopus databases for articles addressing azole resistance in *Aspergillus* spp. isolates from both animals and their immediate environments, published between 2013 and 2024. Descriptive clinical cases were analyzed separately from articles providing *in-vitro* susceptibility test results. MIC_50_ and MIC_90_ values, along with the number of non-wild type (NWT) isolates, were either directly extracted from the articles or calculated based on published results of individual isolates or MIC distributions. Ultimately, seventy-three out of 2042 articles were included in the analysis. Articles reporting clinical cases included only horses, dogs, cats, zoo animals, and wildlife, with the majority of cases occurring outside Europe. Generally, successful clinical remission or recovery followed prolonged and continuous fungicide azole treatments, regardless of the azole-*Aspergillus* spp.-animal category combination. Itraconazole was the most frequently noted treatment in clinical cases involving companion animals (dogs and cats) and horses. The weighted geometric mean of the MIC_50_ values for itraconazole was lowest for *A. fumigatus* isolates within the companion animal category. Zoo animals and wildlife were often treated with voriconazole, and the weighted geometric mean of the MIC_50_ values for this and other azoles was equal to or slightly lower than those calculated for *A. fumigatus* isolates from other animal categories. NWT *A. fumigatus* isolates were reported in zoo animals and wildlife, horses, companion animals, and poultry for several azoles, occurring both in Europe and beyond, in healthy and sick animals. In conclusion, zoo animals and wildlife, horses, and poultry represent a more significant concern regarding the prevalence of *A. fumigatus* and *A. flavus* NWT isolates than other animal categories. Insufficient data prevented conclusions about the situation specifically in Europe, and therefore, more systematic and comparable data are required.

## 1 Introduction

*Aspergillus* species are fungi that are ubiquitous in nature and are known to cause disease in both humans and various animal species. Aspergillosis, an infection caused by *Aspergillus* spp., encompasses a broad spectrum of clinical presentations (1, 2). Among *Aspergillus* spp., *Aspergillus fumigatus* is the most commonly encountered in clinical cases involving humans and animals. Infections can be treated with antifungal azoles, among other options, which in veterinary medicine can be administered both systemically and topically at varying dosages and intervals. The treatment of aspergillosis in animals poses numerous challenges, including prolonged treatment durations (which may therefore be administered intermittently), side effects, differing pharmacokinetics across various animal species, environmental factors, and treatment costs (1). Azole fungicides are registered in Europe solely for use in certain animal species, including cattle, horses, ornamental birds, certain birds of prey, and specific companion and terrarium animals. Consequently, for many other animal species known to be affected by aspergillosis, such as marine mammals, honey bees, and poultry, the treatment and control of aspergillosis present even greater challenges. *In-vitro* susceptibility testing can assist clinicians in developing empirical treatments and adjusting therapy. However, veterinary clinical breakpoints for antifungal drugs to classify *Aspergillus* spp. isolates as susceptible or resistant are currently lacking.

The occurrence of azole resistance among human isolates of *Aspergillus* species, particularly *A. fumigatus*, is rising globally. One contributing factor to this trend is the increased use of azole antifungals in immunocompromised individuals, such as organ transplant recipients, patients in intensive care, and those undergoing long-term corticosteroid treatment. The population at risk for invasive aspergillosis is expanding, with patients often receiving prolonged therapy with broad-spectrum azoles. The clinical impacts of azole resistance are substantial; specifically, in cases of invasive aspergillosis in humans, this resistance is associated with an elevated clinical burden and increased mortality rates (3). The development of resistance to azoles and other antifungal agents in *Aspergillus* species is a complex issue, likely varying among different species. The prolonged administration of azole treatments (patient route) and the extensive use of azole-based fungicides in agriculture (environmental route) are factors associated with genetic mutations that lead to increased resistance through various mechanisms (3, 4). Some *Aspergillus* species can infect both humans and animals, and the treatment of infections in animals often employs similar azole compounds used in human medicine. Moreover, advancements in care and treatment options for companion animals have led to a greater use of azoles in veterinary medicine. Alongside the similar environmental resistance selection presssure observed in both human and animal isolates of *Aspergillus* species, a parallel increase in resistance incidence can be anticipated within veterinary contexts. Current data regarding azole resistance in *Aspergillus* isolates from animals remains sparse and primarily restricted to local studies or individual cases. Talbot et al. (5) did not present evidence of emerging azole resistance among *A. fumigatus* isolates collected from dogs and cats diagnosed with sinonasal aspergillosis (5). Conversely, studies by Ziółkowska et al. (6) and Beernaert et al. (7) reported the identification of resistance to certain azoles in *A. niger* and *A. fumigatus* isolates from avian species, respectively (6, 7).

Recognizing the lack of a global summary regarding the current status of azole resistance in *Aspergillus* isolates relevant to veterinary medicine, this study aimed to determine the occurrence of resistance to fungicide azoles in animal isolates of *Aspergillus* spp. through a systematic literature review.

## 2 Materials and methods

### 2.1 Search strategy

A systematic literature review was conducted to address the research question, “How frequently is resistance to fungicide azoles reported in animal isolates of *Aspergillus* spp.?” To determine the appropriate search strings for the literature review, the following PICO elements of the research question were formulated:

1. Population: *Aspergillus* spp. isolates from various (groups of) animals over the past decade.
2. Intervention: fungicide azoles.
3. Comparison: not applicable.
4. Outcome: resistance.

Based on the pre-formulated PICO question, naive search strings were defined. Following an initial broad search with terms such as ‘animal’, ‘veterinary’, ‘pet’, ‘livestock’, and ‘fauna’ to identify the population, category-specific searches for animals (i.e., zoo animals and wildlife, horses, poultry, and so on) were conducted. Using these initial strings, a calibration exercise was carried out to determine if the output aligned with the research question. Subsequently, search strings were slightly adjusted according to preliminary results. The final searches with the definitive strings (Table 1) took place on 11 March 2024, across both the PubMed and Scopus databases to encompass journals from relevant fields. In PubMed, the query box was utilized, and searches as “All fields.” In Scopus, searches entered in the query box were applied to “ARTICLE TITLE-ABSTRACT-KEYWORDS.” Only articles published from 2013 onwards (including 2013) were selected and uploaded to Rayyan.^1^ Duplicates with a minimum of 97% overlap were automatically removed.

**Table 1:** Search strings used in the final literature search per animal category.

Animal category	Search string
Animals	(“aspergillosis” OR “aspergillus”) AND (“animal” OR “veterinary” OR “pet” OR “livestock” OR “fauna”) AND (“azole” OR “clotrimazole” OR “econazole” OR “econazole nitrate” OR “enilconazole” OR “imazalil” OR “itraconazole” OR “ketoconazole” OR “miconazole” OR “miconazole nitrate” OR “parconazole” OR “posaconazole” OR “fluconazole” OR “voriconazole” OR “fenticonazole” OR “fenticonazole nitrate”) AND (“resistan*” OR “susceptib*” OR “sensitiv*” OR “MIC”)
Zoo animals & wildlife	(“aspergillosis” OR “aspergillus”) AND (“zoo” OR “aquaria” OR “aquarium” OR “aves” OR “bird” OR “duck” OR “mallard” OR “penguin” OR “gull” OR “American crow” OR “corvid” OR “goshawk” OR “eagle” OR “falcon” OR “falconiform” OR “bird of prey” OR “swan” OR “guineafowl” OR “black kite” OR “bustard” OR “kestrel” OR “macaw” OR “parrot” OR “goose” OR “geese” OR “owl” OR “ostrich” OR “partridge” OR “peregrine falcon” OR “pheasant” OR “pigeon” OR “quail” OR “snipe” OR “sparrow” OR “tawny owl” OR “turkey” OR “turtle dove” OR “woodcock” OR “ornamental bird” OR “turaco” OR “budgerigar” OR “cockatiel” OR “cockatoo” OR “common canary” OR “canary” OR “kite” OR “parakeet” OR “starling” OR “rodents” OR “guinea pig” OR “chinchilla” OR “dormouse” OR “dwarf hamster” OR “European hamster” OR “field mouse” OR “gerbil” OR “house mouse” OR “Russian hamster” OR “Syrian hamster” OR “gold hamster” OR “hamster” OR “vole” OR “mouse” OR “small rodent” OR “rat” OR “beaver” OR “bovine” OR “bison” OR “buffalo” OR “Cervidae” OR “reindeer” OR “elk” OR “moose” OR “fallow Cervidae” OR “red dee” OR “roe deer” OR “equine” OR “donkey” OR “zebra” OR “feline” OR “leopard” OR “lion” OR “tiger” OR “wild cat” OR “bobcat” OR “puma” OR “European lynx” OR “lynx” OR “jaguar” OR “camelid” OR “alpaca” OR “llama” OR “camel” OR “canine” OR “racoon dog” OR “wolf” OR “fox” OR “jackal” OR “amphibia” OR “reptile” OR “terrarium animal” OR “tortoise” OR “chuckwalla” OR “marine mammal” OR “aquatic mammal” OR “cetaceans” OR “whale” OR “dolphin” OR “porpoise” OR “pinnipeds” OR “seal” OR “sea lion” OR “walruses” OR “sirenians” OR “manatee” OR “dugong” OR “sea otter” OR “polar bear” OR “fish” OR “rabbit” OR “hare” OR “monkey”) AND (“azole” OR “clotrimazole” OR “econazole” OR “econazole nitrate” OR “enilconazole” OR “imazalil” OR “itraconazole” OR “ketoconazole” OR “miconazole” OR “miconazole nitrate” OR “parconazole” OR “posaconazole” OR “fluconazole” OR “voriconazole” OR “fenticonazole” OR “fenticonazole nitrate”) AND (“resistan*” OR “susceptib*” OR “sensitiv*” OR “MIC”)
Horses	(“aspergillosis” OR “aspergillus”) AND (“equine” OR “horse” OR “ass” OR “asinine” OR “donkey”) AND (“azole” OR “clotrimazole” OR “econazole” OR “econazole nitrate” OR “enilconazole” OR “imazalil” OR “itraconazole” OR “ketoconazole” OR “miconazole” OR “miconazole nitrate” OR “parconazole” OR “posaconazole” OR “fluconazole” OR “voriconazole” OR “fenticonazole” OR “fenticonazole nitrate”) AND (“resistan*” OR “susceptib*” OR “sensitiv*” OR “MIC”)
Small ruminants	(“aspergillosis” OR “aspergillus”) AND (“small ruminant” OR “caprine” OR “ovine” OR “sheep” OR “goat”) AND (“azole” OR “clotrimazole” OR “econazole” OR “econazole nitrate” OR “enilconazole” OR “imazalil” OR “itraconazole” OR “ketoconazole” OR “miconazole” OR “miconazole nitrate” OR “parconazole” OR “posaconazole” OR “fluconazole” OR “voriconazole” OR “fenticonazole” OR “fenticonazole nitrate”) AND (“resistan*” OR “susceptib*” OR “sensitiv*” OR “MIC”)
Companion animals	(“aspergillosis” OR “aspergillus”) AND (“companion animal” OR “pet” OR “amphibia” OR “reptil” OR “reptilia” OR “terrarium” OR “tortoise” OR “chuckwalla” OR “ornamental bird” OR “pigeon” OR “turacos” OR “budgerigar” OR “cockatiel” OR “cockatoo” OR “common canary” OR “cannary” OR “kite” OR “parakeet” OR “chicken” OR “dog” OR “cat” OR “rodent” OR “guinea pig” OR “chinchilla” OR “dormouse” OR “dwarf hamster” OR “European hamster” OR “field mouse” OR “gerbil” OR “house mouse” OR “Russian hamster” OR “Syrian hamster” OR “gold hamster” OR “hamster” OR “vole” OR “mouse” OR “small rodent” OR “rat” OR “alpaca” OR “llama” OR “rabbit”) AND (“azole” OR “clotrimazole” OR “econazole” OR “econazole nitrate” OR “enilconazole” OR “imazalil” OR “itraconazole” OR “ketoconazole” OR “miconazole” OR “miconazole nitrate” OR “parconazole” OR “posaconazole” OR “fluconazole” OR “voriconazole” OR “fenticonazole” OR “fenticonazole nitrate”) AND (“resistan*” OR “susceptib*” OR “sensitiv*” OR “MIC”)
Poultry	(“aspergillosis” OR “aspergillus”) AND (“poultry” OR “chicken” OR “broiler” OR “layer” OR “turkey” OR “duck” OR “ostrich”) AND (“azole” OR “clotrimazole” OR “econazole” OR “econazole nitrate” OR “enilconazole” OR “imazalil” OR “itraconazole” OR “ketoconazole” OR “miconazole” OR “miconazole nitrate” OR “parconazole” OR “posaconazole” OR “fluconazole” OR “voriconazole” OR “fenticonazole” OR “fenticonazole nitrate”) AND (“resistan*” OR “susceptib*” OR “sensitiv*” OR “MIC”)
Bovine	(“aspergillosis” OR “aspergillus”) AND (“bovine” OR “cattle” OR “buffalo”) AND (“azole” OR “clotrimazole” OR “econazole” OR “econazole nitrate” OR “enilconazole” OR “imazalil” OR “itraconazole” OR “ketoconazole” OR “miconazole” OR “miconazole nitrate” OR “parconazole” OR “posaconazole” OR “fluconazole” OR “voriconazole” OR “fenticonazole” OR “fenticonazole nitrate”) AND (“resistan*” OR “susceptib*” OR “sensitiv*” OR “MIC”)
Honey bees	(“aspergillosis” OR “aspergillus”) AND (“honey bee” OR “bee” OR “apiculture” OR “honey”) AND (“azole” OR “clotrimazole” OR “econazole” OR “econazole nitrate” OR “enilconazole” OR “imazalil” OR “itraconazole” OR “ketoconazole” OR “miconazole” OR “miconazole nitrate” OR “parconazole” OR “posaconazole” OR “fluconazole” OR “voriconazole” OR “fenticonazole” OR “fenticonazole nitrate”) AND (“resistan*” OR “susceptib*” OR “sensitiv*” OR “MIC”)

### 2.2 Study eligibility

Two reviewers conducted independent evaluations regarding the potential eligibility of the retrieved articles. To achieve this, the title, abstract, and keywords were meticulously screened, and the following pre-determined criteria were applied: i. The article pertains to animals; ii. The article addresses aspergillosis or *Aspergillus* spp. isolates; iii. The article discusses treatment involving fungicide azoles; iv. The article does not concern animal models for human studies; v. The article does not exclusively emphasize genotypic resistance; vi. The article does not solely describe pharmacokinetic studies; vii. The article is published in English, Dutch, Spanish, French, or German and is a peer-reviewed journal article or other forms of research publication that presents the results of original research published since 2013, satisfying the specified PICO elements and consisting of a full text of over 500 words.

In instances where a reviewer’s decision was merely a “maybe” or when discord existed among the reviewers, the resolution was attained through a systematic discussion of each article, ultimately reaching a consensus regarding its inclusion or exclusion.

### 2.3 Data extraction

All articles submitted for full-text reading were summarized and cataloged in a standard Excel spreadsheet, irrespective of their inclusion or exclusion in the subsequent analysis phase. In this spreadsheet, we documented the characteristics of the publications, encompassing both content-related and descriptive information, such as general details, context, animal host, presence of aspergillosis, and azole use. Additionally, we included study results pertaining to both *in-vitro* susceptibility and treatment outcomes. Publications without access to the full text were excluded at this stage of the study, as were those that did not meet the eligibility criteria upon reviewing the full text.

#### 2.3.1 Study type

Studies were classified as prevalence studies when samples were explicitly collected to evaluate the occurrence of resistance in animal *Aspergillus* spp. isolates. Research involving previously collected samples or isolates was categorized as retrospective studies. Clinical case studies were defined as studies that describe one or more clinical case.

#### 2.3.2 Animal category

In light of the significance associated with the occurrence of aspergillosis and/or the utilization of azoles, the following categories of animals were delineated: zoo animals and wildlife, horses, small ruminants, companion animals, poultry, bovine, honey bees, and others. Given their notable relevance in relation to the occurrence of aspergillosis, horses were designated as a distinct category, separate from companion animals, for the purposes of this study. Specific species within these categories were also documented (for example, “dogs” within the “companion animals” category). Studies examining *Aspergillus* spp. isolates derived from samples obtained from the immediate environments of animals were classified into the corresponding animal category. Findings from publications concerning isolates originating from animals (or their environments) spanning multiple animal categories, or inclusively involving human isolates, were classified under the designation “Several.” In instances where differentiation based on the origin of the isolates was not feasible, results within this category were reported without additional specification.

#### 2.3.3 Animal background

Additionally, isolates were categorized as deriving from healthy animals, sick animals, the environment, a combination of these, or an unknown origin regarding the animals’ condition.

#### 2.3.4 *In-vitro* susceptibility testing results

For studies reporting on *in-vitro* susceptibility testing of *Aspergillus* spp., susceptibility results were categorized according to the type of output from the testing method(s) employed: Minimum Inhibitory Concentration (MIC) (μg/mL), inhibition zone diameter (mm), and growth/no growth on azole-containing agar. Additionally, the categories “other” and “not mentioned” were included. For the “MIC” results, further categorization was conducted based on the testing method(s) used to generate the MIC values: broth microdilution or gradient diffusion.

For publications presenting MIC results, the following data were extracted from the article: the lowest MIC (μg/mL), the highest MIC (μg/mL), the geometric mean (GM) of MICs (μg/mL), MIC_50_ (μg/mL, defined as the MIC at which 50% of isolates were inhibited), and MIC_90_ (μg/mL, defined as the MIC at which 90% of isolates were inhibited). When possible, the number and percentage of tested isolates characterized by MIC values higher than epidemiological cut-off values (ECOFFs) established by the European Committee on Antimicrobial Susceptibility Testing (EUCAST)^2^ were calculated. The MIC_50_ and MIC_90_ values provide insights into the degree of susceptibility of the tested isolates. The number of isolates with a MIC higher than the species-specific ECOFF for a given azole indicates the number of non-wild type (NWT) isolates, i.e. isolates having phenotypically detectable acquired or mutational resistance mechanisms to the azole in question. ECOFFs have been established for the following *Aspergillus* species: *A. flavus*, *A. fumigatus, A. nidulans, A. niger*, and *A. terreus*, concerning isavuconazole, itraconazole, posaconazole, and voriconazole. ECOFFs may only be applied after performing the broth microdilution test as defined by EUCAST. However, to prevent excessive fragmentation of data in further analyses, MIC results obtained via a slightly modified broth (micro)dilution method and gradient diffusion tests were categorized together with those obtained using the EUCAST reference method (EUCAST E.DEF 9.4, March 2022, EUCAST antifungal microdilution method for moulds)^3^ and analyzed accordingly. The data mentioned above were either extracted directly from the publication (when results at the individual isolate level were unavailable) or calculated in Excel (when individual isolate-level results or MIC distributions were available). When calculating the geometric mean of MIC values, MIC_50,_ and MIC_90_, MIC values equal to or lower than the lowest azole concentration in the test range were set at the lowest tested concentration (e.g., ≤0.125 μg/mL was regarded as 0.125 μg/mL). Similarly, MIC values exceeding the highest azole concentration in the test range were set at the highest tested concentration (e.g., >16 μg/mL was regarded as 16 μg/mL).

For publications reporting inhibition zone diameters, the following data were extracted from the article: smallest inhibition zone diameter (mm), largest inhibition zone diameter (mm), and the geometric mean of inhibition zone diameters (mm). Either this data was copied from the publication (in the absence of results at the individual isolate level) or calculated using Microsoft Excel (when results at the individual isolate level were available). No ECOFFs have been established by EUCAST for assessing the susceptibility of *Aspergillus* to azoles based on disk diffusion. Consequently, the number and percentage of NWT isolates could not be calculated, and disk diffusion results were not analyzed in further detail.

When only one *Aspergillus* isolate of a particular species or section was tested, the susceptibility result was placed in the column with geometric mean data (mm or μg/mL), and the remaining susceptibility result columns were left empty (i.e., the lowest and highest values, and MIC_50_ and MIC_90_, if applicable).

When multiple susceptibility testing methods were employed in a study (e.g., disk diffusion and MIC-based methods), the results obtained from different methods were analyzed separately.

### 2.4 Statistical analysis

Further data validation and descriptive analyses were conducted in Stata version 17.0. Regarding the *in-vitro* susceptibility data, based on the MIC_50_ values of isavuconazole, itraconazole, posaconazole, and voriconazole determined for *A. fumigatus* isolates in various studies, the weighted geometric mean (GM) of the MIC_50_ values for each of these azoles was calculated. The weighted GM represents the GM of all MIC_50_ values found in the studies, weighted by the number of isolates in those particular studies (analytical weights). Similarly, the weighted GM of the MIC_50_ values for isavuconazole, itraconazole, posaconazole, and voriconazole, determined for all *Aspergillus* spp. isolates described in the studies, was calculated. Figures were created using Datawrapper^4^.

## 3 Results

### 3.1 General results

After applying the search strings, 2042 articles were found in total: 462 in the PubMed database and 1,580 in the Scopus database.

**Figure 1:**
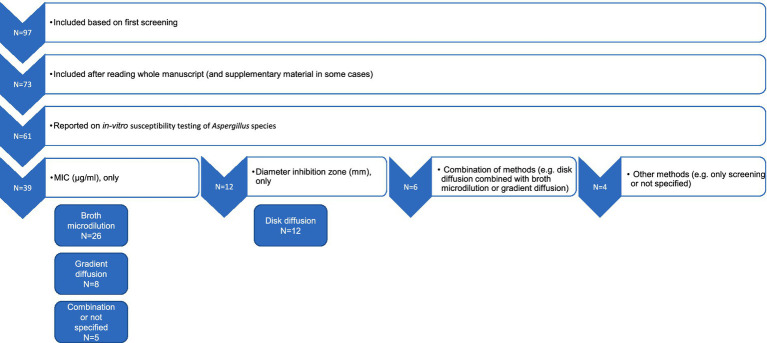
A schematic flowchart illustrating the selection of the articles and processing of *in-vitro* susceptibility data for further analyses. The numbers indicate the number of articles reporting different susceptibility results obtained using a combination of various testing methods.

After eliminating duplicates and conducting an initial screening of titles, abstracts, and keywords, 97 articles were included for full-text review (Figure 1). Of these, 73 were ultimately included in the review, while 24 were excluded following a detailed evaluation. Reasons for exclusion included lack of access to the complete manuscript, absence of data on *in-vitro* susceptibility of *Aspergillus* spp. to azoles or treatment outcomes, absence of data on *Aspergillus* spp. or azoles, difficulties in retrieving and interpreting the presented data, or review articles that reported results from several original research studies. Each original study referenced in the review articles was individually verified. This led to the inclusion of one additional article, which was previously not part of the original search (8), that met the eligibility criteria. Articles published after 2013 that described studies conducted before 2013 were included. Clinical cases in which azole susceptibility testing was not carried out but where animals affected by *Aspergillus* spp. received treatment were included and described separately from the *in-vitro* susceptibility results.

**Figure 2:**
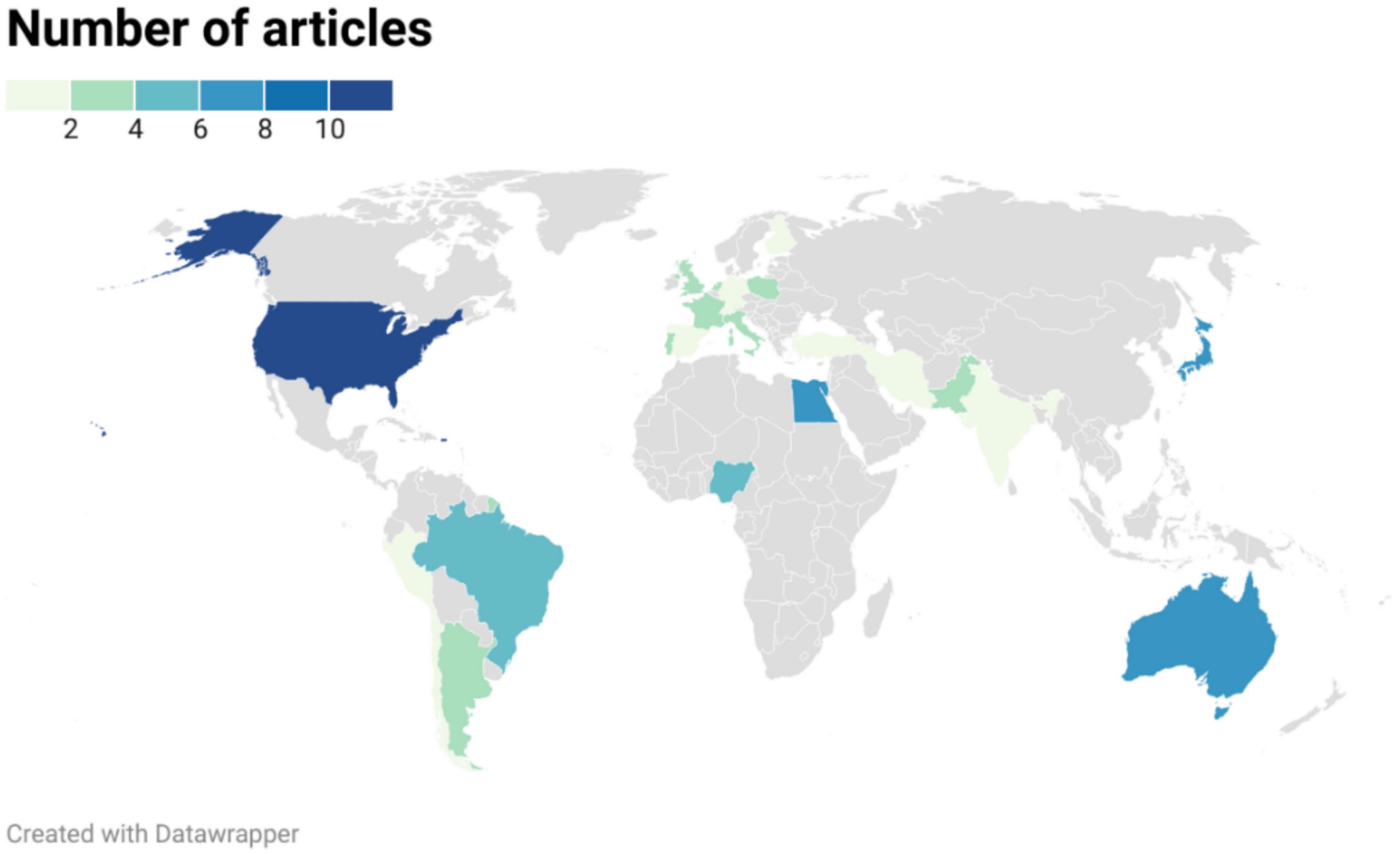
Distribution of geographical study locations for all included publications regarding the resistance of *Aspergillus* spp. to azoles (*N* = 73).

The articles included in the final review were published between 2013 and 2024, showing a peak from 2019 to 2022. The majority of the studies originated from non-European countries (Figure 2).

The majority of the studies (42.5%) were classified as prevalence studies, followed by clinical cases and retrospective studies (31.5 and 26.0%, respectively). Regarding animal categories, the majority of studies focused on companion animals (33%) and zoo animals and wildlife (26%). Horses (15%) and poultry (12%) were also commonly mentioned. Among the remainder, there were studies on bovine, honey bees, and others.

### 3.2 Clinical cases

The clinical cases (*N* = 23) were analyzed separately from articles providing *in-vitro* susceptibility testing results. Two articles described individual cases of aspergillosis in horses (9, 10), whereas five articles detailed cases of aspergillosis in animals classified as zoo animals and wildlife, although some were kept as companion animals (11–15). In the two horse cases, *A. fumigatus* was isolated, and treatment with itraconazole alongside supportive therapy was administered for 45 days. One horse appeared to recover, while the other showed improvement in clinical signs, although no subsequent records were available. The five cases involving zoo animals and wildlife comprised five different species, with various treatments resulting in different outcomes. The only European case involved a bottlenose dolphin from the Netherlands that developed pulmonary aspergillosis following prolonged antibiotic therapy, with cultures yielding *A. fumigatus*. Based on susceptibility testing results, oral treatment with voriconazole was switched to posaconazole three months after initiation of the treatment due to lack of clinical improvement. After more than 14 months of treatment, a complete clinical resolution was reported (11). The remaining clinical cases (*N* = 16) involved cats and dogs. Some articles (16–18) concerning these cases did not describe treatment protocols or outcomes but provided susceptibility results for different azoles; therefore, they were considered solely for the analyses of *in-vitro* susceptibility results. Regarding clinical cases in cats (19–22), although various *Aspergillus* spp. were identified (*A. udagawae, A. viridinutans,* and *A. fischeri*), four out of five cases were treated systemically with itraconazole; in one instance, this was combined with locally applied clotrimazole, and in two instances, treatment followed surgical debridement of the infection site. The remaining case did not receive treatment. Of the four treated cats, only one showed apparent remission after four months of oral itraconazole treatment. In the other cases, treatment was either too short or applied intermittently. None of the mentioned cases of cats occurred in Europe. As for the cases in dogs (8, 23–30), only three articles reported cases from Europe—one from Italy and two from England. Several different *Aspergillus* spp. were isolated, including *A. terreus, A. caninus, A. fumigatus, A. niger*, and *A. versicolor*, and various treatments with differing outcomes were documented. Corrigan et al. (23) described ten cases of dogs with disseminated aspergillosis, all treated with the same protocol of posaconazole via oral administration, to assess the safety and efficacy of this approach. This treatment was not employed in any other dog case, suggesting that, although approved in Europe, posaconazole does not appear to be commonly used based on these findings. In the study by Corrigan et al. (23), only one of the ten cases seemingly recovered. The remaining nine cases exhibited some temporary remission or improvement but ultimately relapsed, with one case lost to follow-up. Treatments in the dog cases included itraconazole, voriconazole, clotrimazole, fluconazole, and ketoconazole. Among those treated with itraconazole, one case that received oral treatment and topical ketoconazole in the ear showed apparent recovery. Two cases receiving itraconazole for at least three months exhibited noticeable remission, while three did not. Of the last three, in two cases susceptibility results showed a higher susceptibility of the isolate to voriconazole; however, this option was not pursued due to financial constraints or side effects. The other case remained stable, with no remission of clinical signs observed. One case treated with topical voriconazole in conjunction with keratectomy showed improvement, although no follow-up records regarding the effects after treatment cessation were available. Another case treated with voriconazole did not demonstrate remission of clinical signs after 11 months of treatment. In two cases treated with clotrimazole following sinusotomy, one case experienced clinical remission but ultimately died due to other causes, whereas the other did not show remission. Of the two cases treated with fluconazole, one was lost to follow-up shortly after treatment initiation, while the other did not demonstrate remission after one month. A more detailed description of the clinical cases is provided in Supplementary Tables 1, 2.

### 3.3 *In-vitro* susceptibility testing results

Of the 73 articles included, 61 reported on *in-vitro* susceptibility testing of *Aspergillus* spp. (Figure 1). The methods documented in these publications primarily consisted of broth microdilution, gradient diffusion, and disk diffusion. In some studies, combinations of these methods were employed, while in others, different methods were used or not specified at all.

In 39 studies, only MIC values were determined, either through broth microdilution alone (*N* = 26) or gradient diffusion alone (*N* = 8). In five studies, a combination of MIC-based susceptibility testing methods was employed, or the MIC method was not fully specified. Six studies utilized a mixture of different methods, producing various types of output, such as both broth microdilution and gradient diffusion or both gradient and disk diffusion. Only disk diffusion was used in 12 articles. Four studies adopted alternative methods (for example, only screening *Aspergillus* isolates or samples for azole resistance) or did not specify the method employed. For screening the presence of azole-resistant *Aspergillus* spp., *Aspergillus* isolates or samples were inoculated onto azole-supplemented agar, and growth on this azole-containing agar was considered indicative of the presence of azole-resistant isolates (31–36) (Supplementary Table 3). Some studies subsequently tested the isolates using the broth microdilution reference method to confirm the presence of resistant *Aspergillus* isolates (Supplementary Table 4).

A total of 29 studies reported MIC values for *A. fumigatus* isolates (5, 6, 10, 11, 31–34, 37–57), while 10 studies reported values for *A. flavus* isolates (12, 38, 40, 41, 45, 46, 49, 50, 53, 55). Further analyses were primarily conducted on these two *Aspergillus* species in conjunction with the azoles isavuconazole, itraconazole, posaconazole, and voriconazole. MIC results for these two *Aspergillus* species are summarized in Supplementary Tables 4, 5, respectively, categorized by animal category. MIC results for other, less frequently reported *Aspergillus* species and isolates that are not further identified at the species level but belong to the genus *Aspergillus* or a specific section of *Aspergillus* (5, 6, 10, 13, 16, 17, 21, 22, 24, 29, 37, 40, 41, 44–47, 49–65) are summarized in Supplementary Table 6. These results are collectively presented in one table, as no ECOFFs could be applied for further interpretation of the MICs, and the *Aspergillus* species-specific results per animal category were too few to present in a separate table (which applies to the *A. nidulans*, *A. niger*, and *A. terreus* isolates tested).

**Figure 3:**
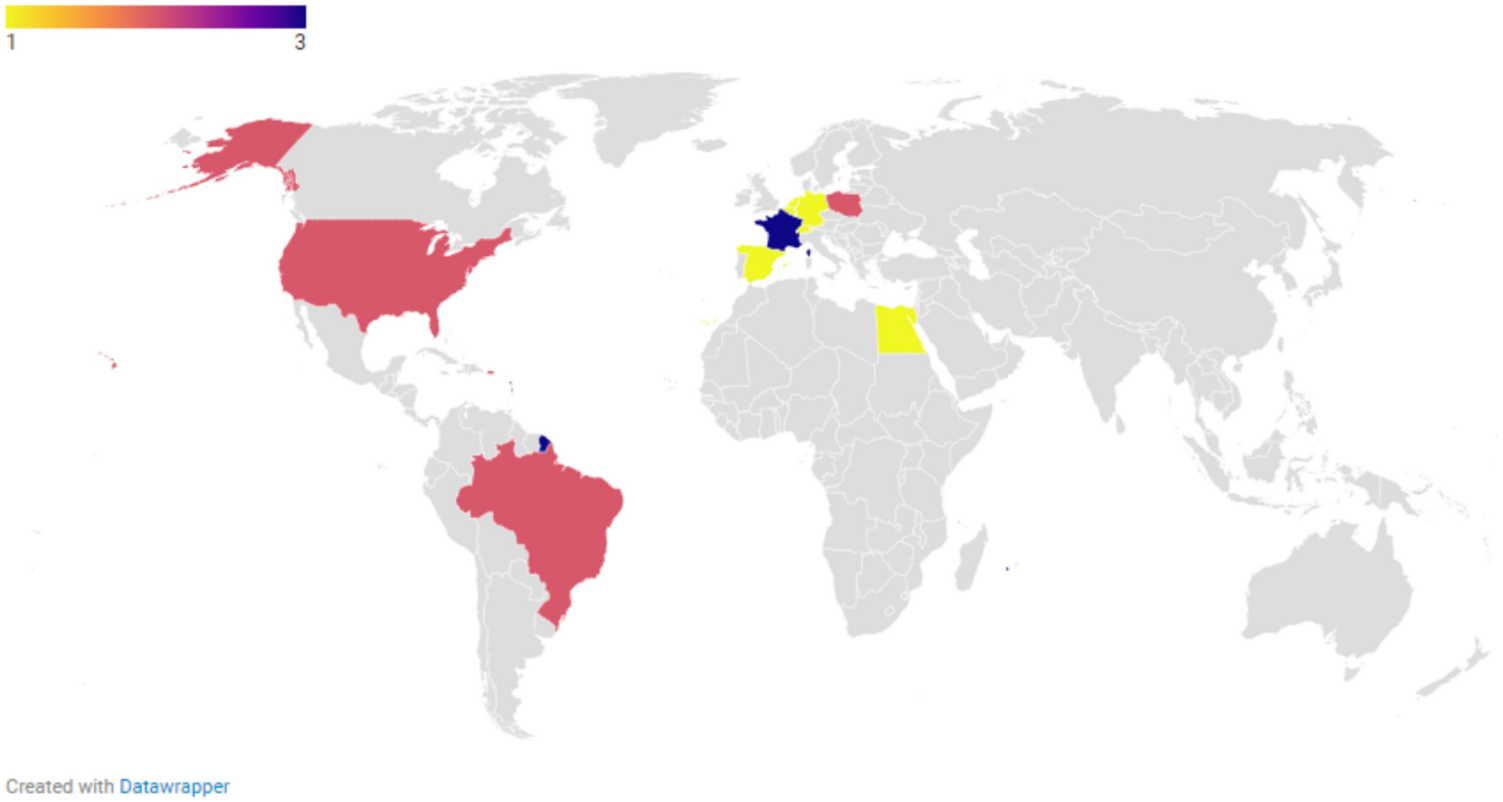
The number of unique studies reporting at least one isolate with a minimum inhibitory concentration (MIC) value exceeding the species-specific epidemiological cut-off (ECOFF) for any of the following azoles: itraconazole, posaconazole, voriconazole, and isavuconazole.

**Figure 4:**
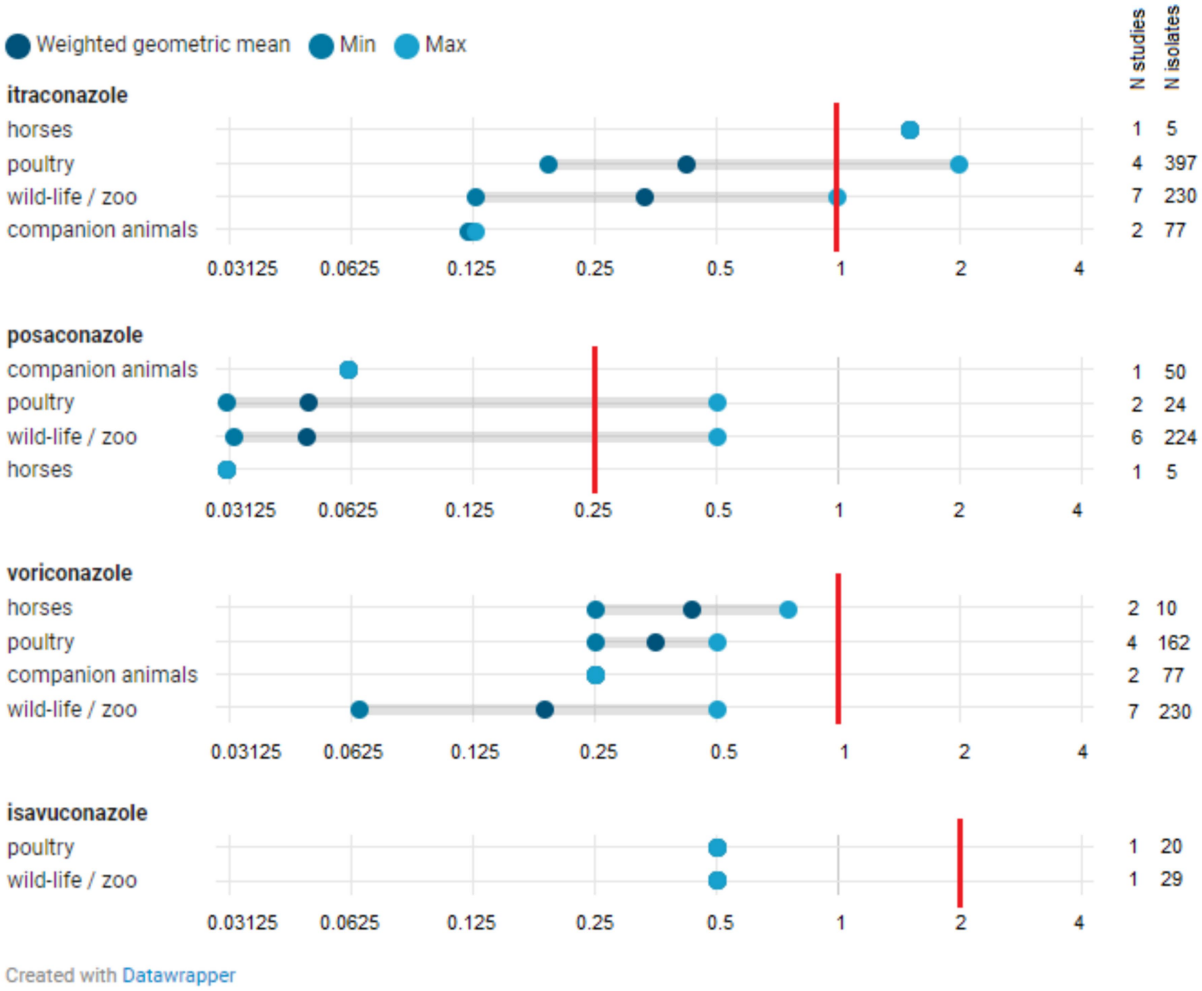
Distribution of MIC_50_ for *Aspergillus fumigatus* isolates. The minimum MIC_50_ identified in one study is indicated, along with the maximum MIC_50_ reported in another study. The weighted geometric mean represents the geometric mean of all MIC_50_s found across studies, adjusted for the number of isolates in those specific studies (analytical weights). The vertical red line indicates the epidemiological cut-off (ECOFF). The MIC_50_ denotes the minimum inhibitory concentration (MIC) at which 50% of isolates are inhibited.

**Figure 5:**
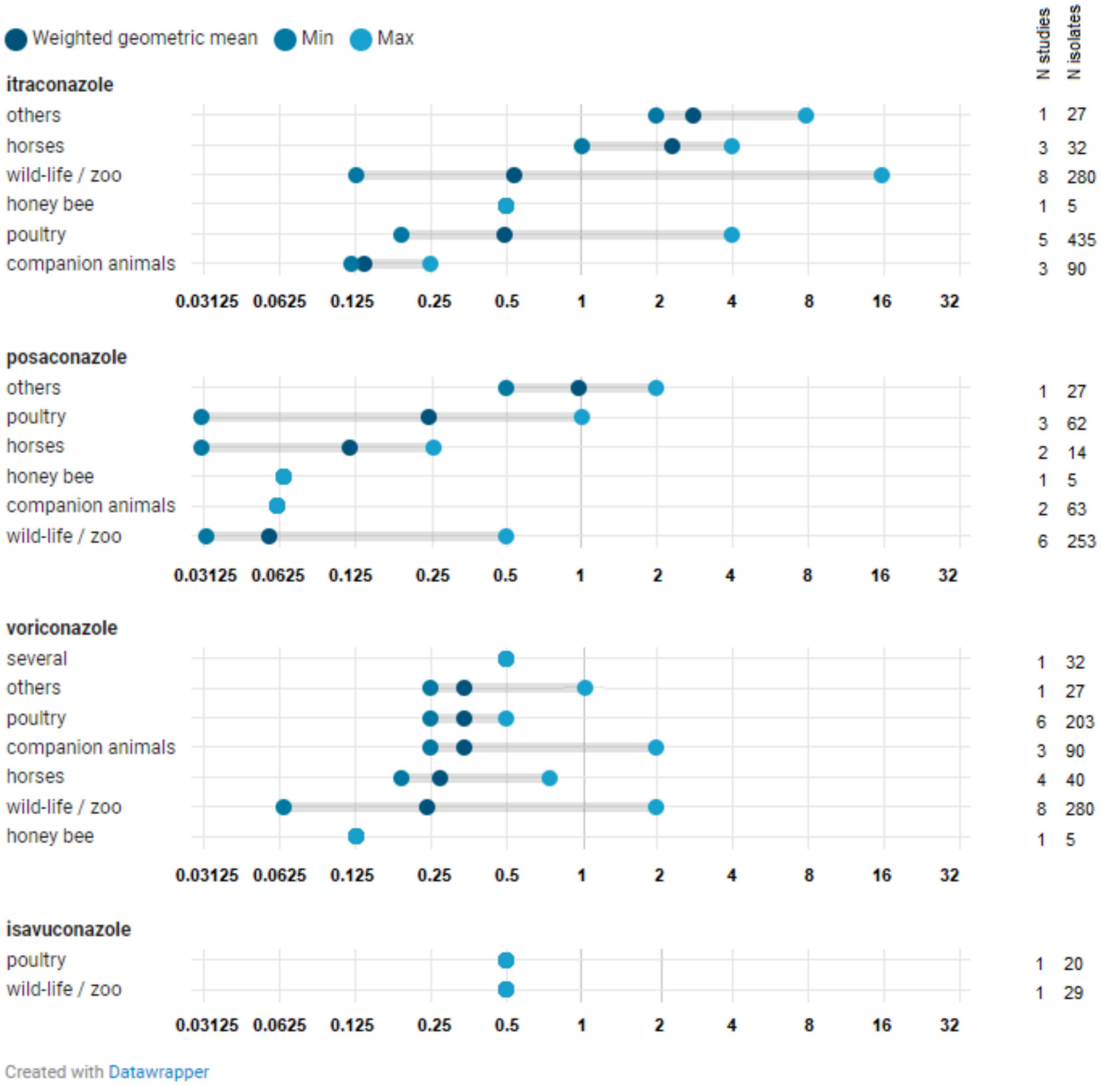
Distribution of MIC_50_ for all *Aspergillus* spp. isolates. The minimum MIC_50_ reported in a study for a specific *Aspergillus* species is indicated, along with the maximum MIC_50_ observed in a study for that species. The weighted geometric mean represents the geometric mean of all MIC_50_ values identified in studies, adjusted based on the number of isolates in those particular studies (analytical weights). The MIC_50_ refers to the minimum inhibitory concentration (MIC) at which 50% of isolates are inhibited.

The number of studies reporting at least one *Aspergillus* isolate above the species-specific ECOFFs (i.e., NWT isolates that have acquired resistance) was limited (*N* = 18) and included studies from Europe as well as those from outside Europe (Figure 3; Supplementary Tables 4–6). Figures 4, 5 show the lowest and highest MIC_50_ values and the weighted GM of the MIC_50_ values calculated from the findings of the articles for *A. fumigatus* isolates and all *Aspergillus* spp. isolates described in the studies, respectively.

*A. fumigatus* isolates with a MIC value exceeding the ECOFF have been reported in zoo animals and wildlife, horses, companion animals, and poultry across one or more of the four azoles (isavuconazole, itraconazole, posaconazole, and voriconazole), both within Europe and beyond, and in both healthy and sick animals (Supplementary Table 4). The weighted GMs of the MIC_50_ values for isavuconazole, itraconazole, posaconazole, and voriconazole, calculated for each animal category, were all below the respective ECOFFs (Figure 4). This indicates that the growth of half of the *A. fumigatus* isolates tested in these studies was inhibited by a concentration lower than the ECOFF, thus categorizing them as part of the susceptible wild-type (WT) population. Furthermore, the weighted GM of the MIC_50_ values did not significantly differ across *A. fumigatus* isolates from various animal categories. The lowest weighted GMs of the MIC_50_ values were observed for posaconazole.

Fewer studies reported findings on azole susceptibility in *A. flavus* isolates (Supplementary Table 5). NWT isolates were reported regarding susceptibility to itraconazole, posaconazole, and voriconazole in horses and in zoo animals and wildlife, both in Europe and beyond.

The MIC_50_ values of posaconazole, voriconazole, and isavuconazole found for other *Aspergillus* species and isolates that are not classified at the species level (see Supplementary Table 6) were not significantly different from those observed for *A. fumigatus* and *A. flavus*. The same applies to the itraconazole values. However, the MIC_50_ values of itraconazole for isolates from zoo animals and wildlife appear to be somewhat higher for the other *Aspergillus* species tested (see Supplementary Table 6).

The results of testing the susceptibility of *Aspergillus* isolates using disk diffusion (6, 18, 30–32, 34–36, 52, 66–75) are briefly summarized in Supplementary Table 7. Where available, inhibition zone diameters are provided, along with some conclusions drawn by the respective research groups.

Supplementary Table 3 presents data from studies that used a screening method to detect azole resistance in *Aspergillus* isolates, employing azole-containing agar plates. One of the cited studies applied this method to air samples, examining them for the presence of resistant *Aspergillus* spp., which were subsequently identified as *A. fumigatus*. The outcomes of these screening studies, which typically tested larger numbers of isolates and samples, indicated that the prevalence of azole-resistant *Aspergillus* isolates varied considerably and depended on the origin of the isolates and samples. Resistance was primarily observed in isolates and samples from zoo animals and wildlife, or their environments (0.5–31%).

## 4 Discussion

In this study, we collected and analyzed data on the occurrence of (phenotypic) azole resistance in *Aspergillus* spp. isolates from animals across various countries over the last decade through a systematic literature review. Due to feasibility and access permission constraints, only two databases were used for the searches: PubMed and Scopus. This may lead to the omission of other relevant articles found in different databases or grey literature, such as the study by Das et al. (76). The country, year of study, animal background, and methodology varied significantly among studies, making the comparison, integration, and analysis of results challenging. According to this review, the primary animal categories for which results on azole susceptibility of *Aspergillus* spp. isolates were reported include poultry, companion animals (mainly dogs and cats), zoo animals and wildlife, and horses. Among European countries, studies conducted in Denmark, England, Finland, France, Germany, Ireland, Italy, the Netherlands, Poland, Portugal, Spain, and Switzerland met the eligibility criteria. Outside of Europe, the highest number of eligible studies was carried out in the United States, Australia, and Egypt. The number of publications ultimately selected for full-text reading was surprisingly low, given that the inclusion criteria encompassed a ten-year time frame, non-country restrictions, and all relevant animal species. Additionally, publications may be inevitably biased by the inclination to publish only positive results, while negative results are often overlooked or not reported. Consequently, aside from the previously mentioned challenges, the outcomes of this literature review may overestimate the clinical significance of azole resistance in animal *Aspergillus* spp. isolates compared to other animal health issues.

In veterinary medicine, and often in human medicine as well, the selection of a fungicide for treating aspergillosis is typically grounded in experience. As a result, the articles detailing clinical cases were included to utilize the clinical outcomes following azole treatment as a means of evaluating the susceptibility of *Aspergillus* spp. isolates to the azole used in the treatment. However, the treatment of aspergillosis is rarely based solely on azole therapy. Regardless of the category of animal, treatment generally involves a combination with supportive therapy or surgical excision of masses. These masses are often situated around the eyes (retrobulbar, orbital, or keratomycosis), nose, or sinuses (nasal or sinonasal). Furthermore, azoles have been noted to be administered orally, intravenously, or via indwelling at the site of the mass. In several instances, adherence to the treatment could not be guaranteed, or follow-up was insufficient. These factors may all have impacted the clinical outcomes in these case reports, making it difficult to ascertain whether the azole administered was effective against the relevant *Aspergillus* spp. causing the infection. Generally, cases reporting remission of clinical signs or recovery shared a commonality of prolonged and continuous treatments, irrespective of the azole-*Aspergillus* spp.-animal category combination.

Regarding the *in-vitro* susceptibility results, various testing methods were employed to assess susceptibility to azoles. Furthermore, the interpretation criteria applied were not always clearly defined. In recent years, significant efforts have been made to optimize and standardize *in-vitro* susceptibility testing of *Aspergillus* spp. to azoles, aiming to reliably detect resistance and to minimize inter- and intra-laboratory variation. Key factors in MIC-based susceptibility testing of *Aspergillus* isolates include macro versus microdilution, growth media, drug dilutions, inoculum size, incubation temperature and time, and reading method (77). The majority of studies utilizing broth microdilution referred to either the reference method of the Clinical & Laboratory Standards Institute (CLSI), CLSI M38, or the EUCAST reference method (EUCAST E.DEF 9.4 March 2022 EUCAST antifungal microdilution method for moulds)^5^. However, commercial broth microdilution tests were also used. Other studies employed gradient diffusion to determine MICs. For interpreting MIC results, studies either referred to CLSI or EUCAST, while a few cited relevant literature. There are no veterinary breakpoints for classifying *Aspergillus* isolates as susceptible or resistant; thus, either human clinical breakpoints or ECOFFs (if available) were utilized for the interpretation of MIC values in these studies. The challenges associated with establishing clinical breakpoints in animals for *Aspergillus* spp. may be linked to a highly variable bioavailability of azoles across different animal species or the absence of registered azoles in veterinary medicine. Consequently, interpreting the MICs to guide therapeutic choices in the absence of veterinary (animal-specific) clinical breakpoints should be approached with caution. Nonetheless, MIC values still provide valuable information regarding susceptibility, particularly when ECOFFs exist for the specific combination of *Aspergillus* spp. and azole to differentiate WT and NWT isolates. Broth microdilution susceptibility testing is time-consuming and requires well-trained personnel. Therefore, an easy-to-use reference screening method for detecting *Aspergillus* isolates with potential azole resistance has been developed (EUCAST E.Def 10.2 June 2022 EUCAST agar screening for resistance in *Aspergillus* spp.). The principle of this screening test is based on preparing plates containing agars supplemented with itraconazole and voriconazole, with or without posaconazole, alongside a drug-free control agar. After solidifying the agar, the plates are inoculated with a standardized *Aspergillus* inoculum on the surface of each agar. Following 48 h of incubation, *Aspergillus* growth is visually assessed. Any growth on one or more azole-containing agars should be taken into account and confirmed by MIC testing. Several studies employing such a screening method referred to the EUCAST reference method, while others cited literature. The use of diverse test methods and interpretation criteria, the lack of ECOFFs, and the fact that the isolates tested may not have been randomly selected (e.g., being tested following a positive screening result using azole-containing agars or originating from animals with different clinical backgrounds) hindered the analysis and necessitated careful interpretation of the *in-vitro* data analyses. Azole resistance can also be detected by genotypic methods (not included in the current study). A limitation of genotypic detection of resistance is that molecular tests can confirm resistance but not susceptibility.

Itraconazole was the most commonly used azole noted in the studied cases involving dogs, cats, and horses, occasionally combined with other fungicide azoles, such as clotrimazole or ketoconazole. Publications reporting susceptibility results of *A. flavus* isolates in companion animals were not found. Based on the data analyzed in this review, the weighted GM of the MIC_50_ values (as a measure of the degree of susceptibility of the tested isolate population) of itraconazole for *A. fumigatus* isolated from companion animals was lower than the values obtained for *A. fumigatus* isolates from other animal categories. This indicates a high degree of susceptibility of *A. fumigatus* isolates from dogs to itraconazole. Additionally, the weighted GM of the MIC_50_ values of itraconazole for all *Aspergillus* spp. from companion animals was lower than those calculated for other animal categories. Unfortunately, in several clinical cases describing itraconazole treatments, the completion of treatment was often not achieved (either too short or inconsistent). In cases where treatment was appropriately administered (mainly in dogs), clinical signs were frequently remitted, or the animals appeared to recover (27–29). Taking into account both the clinical and *in-vitro* susceptibility results, we can conclude that itraconazole is a suitable option for the treatment of aspergillosis in companion animals, particularly for *A. fumigatus* infections. Comparing cases in zoo animals and wildlife proved challenging due to the varied species affected (including dolphins, cormorants, ducks, Okinawa rail, and wallabies), different *Aspergillus* spp. isolated (*A. allahabadii*, *A. fumigatus*, and *A. flavus*), and the use of different azoles (fluconazole, voriconazole, and posaconazole). In general, four out of the five clinical cases of zoo animals and wildlife included in this systematic review were treated with voriconazole. This azole is currently registered for human use only and not for veterinary use in Europe. The absence of registered azole-based fungicidal products and treatment guidelines for this animal category might result in off-label use of these products, increasing the risk of unsuccessful clinical outcomes. Considering the susceptibility results for the *Aspergillus* spp. isolated in this animal category, isolates characterized by MIC values exceeding specific ECOFF values were found among *A. fumigatus,* indicating reduced susceptibility to itraconazole, posaconazole, voriconazole, and isavuconazole (seven, three, four, and two studies, respectively) (11, 31–34, 39, 42). One study reported a NWT *A. flavus* isolate with acquired resistance to voriconazole (12). However, the weighted GM of the MIC_50_ values for these azoles was equal to or slightly lower than those calculated for *A. fumigatus* and all *Aspergillus* spp. isolates from other animal categories, particularly those from horses (excluding the weighted GM of the MIC_50_ values of itraconazole for *A. fumigatus* and all *Aspergillus* spp. isolates from companion animals, which was somewhat lower). These results imply that zoo animals and wildlife may represent a significant category regarding frequent off-label use and the occurrence of NWT isolates. A recent study published by Das et al. in 2023 (not included in our searches) on the resistance of various azoles in *Aspergillus* spp. isolated from falcons reported an apparent increase in MIC values for posaconazole, voriconazole, and itraconazole in recent years (76).

In the studies included in this review regarding horses, both *A. fumigatus* and *A. flavus* isolates exhibiting acquired resistance to itraconazole, posaconazole, and voriconazole were reported (single studies). The MIC values for *Aspergillus* spp. combined were among the highest for itraconazole, the lowest for posaconazole, and the voriconazole MIC values corresponded with those reported for *Aspergillus* spp. from other animal species. Interestingly, the two studies detailing clinical cases in horses indicated an improvement in clinical signs after 45 days of itraconazole treatment in conjunction with other therapies. However, follow-up on the patients in these two cases is lacking, making it difficult to ascertain whether these results align with or contradict the susceptibility data for *A. fumigatus* isolates. Given the documented presence of *A. fumigatus* and *A. flavus* isolates with acquired resistance to itraconazole, the choice of this treatment should be re-evaluated in countries where it is (apparently) frequently administered.

Despite the apparent absence of azoles as treatments in poultry production and the lack of authorized azoles for chicken, turkeys or geese in Europe, NWT *A. fumigatus* isolates from poultry and their immediate environment have been reported with regard to susceptibility to itraconazole (several studies), posaconazole (one study), and voriconazole (one study). These findings suggest that resistance in poultry isolates may have been acquired through the environmental rather than the patient route. Considering the data analyzed in this review, the MIC values for all *Aspergillus* spp. combined were not significantly higher for isolates from poultry compared to those reported for other animal categories.

The occurrence of resistance in human *A. fumigatus* isolates has increased in recent years, and infections in human patients with azole-resistant isolates acquired from the environment have recently been documented (78). The results from our study detail the presence of azole-resistant *A. fumigatus* isolates in various animal species. Both human, veterinary, and agricultural azole fungicides target the same molecular site in *A. fumigatus* spp., which is frequently implicated in the acquisition of resistance (79). Therefore, a comparable risk of azole-resistance acquisition from the environment may be anticipated in animal isolates. However, data on this issue are scarce, as is the potential risk posed to public health by the use of azole fungicides in veterinary medicine. Future research should focus on the mechanisms and epidemiology of resistance within a One Health context, as well as on developing new antifungal strategies to address this challenge.

## 5 Conclusion

This study provides a contemporary cross-country overview of the susceptibility of *Aspergillus* spp. isolates from animals or their direct environment to commonly used azoles in veterinary medicine. The complexity of interpreting and integrating results from non-uniform studies regarding population size, background, methodology, and interpretation criteria limits the ability to draw valid conclusions. Overall, it can be concluded that zoo animals and wildlife, horses, and poultry represent a greater concern regarding the occurrence of *A. fumigatus* and *A. flavus* NWT isolates compared to other animal categories. The suspected co-existence of a patient and environmental route, based on these findings, supports the necessary One Health approach of monitoring and controlling the emergence of azole resistance. More systematic and comparable data are required to accurately assess the prevalence of azole-resistant *Aspergillus* isolates, particularly in Europe, and to evaluate trends over time and differences among countries or animal categories. Furthermore, in addition to accurately identifying the *Aspergillus* spp. responsible for the infection, there is an urgent need for species-specific clinical breakpoints to classify strains as susceptible or resistant to inform therapeutic decisions.

## Data Availability

The original contributions presented in the study are included in the article/Supplementary material, further inquiries can be directed to the corresponding author.

## Footnotes

^1^ https://rayyan.ai

^2^The European Committee on Antimicrobial Susceptibility Testing. Overview of antifungal ECOFFs and clinical breakpoints for yeasts, moulds and dermatophytes using the EUCAST E.Def 7.4, E.Def 9.4 and E.Def 11.0 procedures. Version 4.0, 2023. http://www.eucast.org.

^3^ https://www.eucast.org/astoffungi/methodsinantifungalsusceptibilitytesting/ast_of_moulds

^4^ https://www.datawrapper.de

^5^ https://www.eucast.org/astoffungi/methodsinantifungalsusceptibilitytesting/ast_of_moulds
