# Supplementary material for: Azole resistance in Aspergillus isolates from animals or their direct environment (2013–2023): a systematic review
Source: Front Vet Sci. 2025 Mar 20;12:1507997. doi: 10.3389/fvets.2025.1507997 (PMC11967370; doi:10.3389/fvets.2025.1507997)
Supplement: Supplementary file 1 [file Table_1.docx]

**Supplementary Table 1**. Summary of clinical cases of aspergillosis in cats where treatment protocol and outcome are described in the respective articles.

| Reference | Country | Clinical presentation | *Aspergillus* spp*.* | Treatment and outcome | Interpretation |
| --- | --- | --- | --- | --- | --- |
| Bartels *et al*., 2022 | USA | Right ventral cervical mass | *Aspergillus* spp*.* | Excision of the mass and lymph node and inconsistent treatment with liquid suspension **itraconazole** (5.05mg/kg via oral q24h) together with anti-inflammatory and pain-relief medication. Relapse after 5 months and again excision and similar treatment again inconsistently applied. Relapse 11 months later. Due to the extension of the fungal granuloma and the recurring nature, the cat was humanely euthanized. | Azole treatment inconsistently applied. Difficult to assess effect. |
| da Costa *et al*., 2019 | Brazil | Retrobulbar mass | *Aspergillus* spp*.* | After enucleation of the left eye, the cat was treated with intranasal **clotrimazole** once a week and systemic **itraconazole** (50 mg via oral per day). Due to recurring of a mass in the palate and clinical worsening the cat was treated with subcutaneous amphotericin B and supportive therapy. However, the cat died two days after initiation of the treatment. | Acute death and therefore too short treatment. Difficult to assess effect. |
| Kano *et al*., 2013 | Japan | Orbital aspergillosis | *A. udagawae* | High-dose of **itraconazole** treatment (50 mg/dose via oral twice a day ) was applied for 3 months. Two months after treatment, the mass was again detected and the treatment re-initiated. One month after re-initiation of the treatment, no more clinical signs and the mass was not again visually detected. | Apparent remission. |
| Kano *et al*., 2013 | Japan | Orbital aspergillosis | *A. viridinutans* | The owner refused to treat the animal, the conditioned worsened and the cat died 10 days later due to sarcoma and aspergillosis. | No treatment, no assessment of effect. |
| Kano *et al*., 2015 | Japan | Nasal cavity mass | *A. fischeri* | **Itraconazole** was administered (10 mg/kg, via oral once a day) for 12 days. The owner refused intranasal treatment waiting for susceptibility results. Clinical conditions worsened and the cat died 28 after initiation of the treatment. | Systemic itraconazole, short duration, no remission. |
