# Supplementary material for: Azole resistance in Aspergillus isolates from animals or their direct environment (2013–2023): a systematic review
Source: Front Vet Sci. 2025 Mar 20;12:1507997. doi: 10.3389/fvets.2025.1507997 (PMC11967370; doi:10.3389/fvets.2025.1507997)
Supplement: Supplementary file 2 [file Table_2.docx]

**Supplementary Table 2**. Summary of aspergillosis clinical cases in dogs where treatment protocol and outcome are described.

| Reference | Country | Clinical presentation | *Aspergillus* spp*.* | Treatment and outcome | Interpretation |
| --- | --- | --- | --- | --- | --- |
| Nevile, Hurn and Turner, 2016 | Australia | Keratomicosis (with corneal ulcer) | *Aspergillus* spp*.* | Conservative keratectomy and 360-degree conjunctival graft were performed followed by topical antibiotic and atropine treatment and oral anti-inflammatory medication combined with topical (eye drops) **voriconazole** six times daily. Thirty-two days after surgery, clinical signs improved and treatment was discontinued. | No records after discontinuation of treatment. Apparent remission together with keratectomy. |
| Pilkington *et al*., 2022 | England | Sinonasal aspergillosis | *Aspergillus* spp*.* | After frontal sinusotomy was performed, **itraconazole** (6.7 mg/kg, via oral q 12 h) for 3 months was applied. Clinical signs improved, but returned when cessation of itraconazole treatment. Therefore itraconazole was prolonged for 4.5 more months and after that period the dosage was tapered by 25% each month. At the last control, 311 days after the diagnosis, clinical signs resolved and the itraconazole treatment was ended one month later (3.3 mg/kg, via oral, q 24 h for 1 month). | Apparent remission. |
| Smith *et al*., 2018 | Australia | Fungal rhinitis | *Aspergillus* spp*.* | Treatment with **itraconazole** (2.5 mg/kg via oral twice daily) for 3 months was applied. Five months later, the clinical signs resolved. | Apparent remission. |
| Corrigan *et al*., 2016 | USA | Disseminated aspergillosis | *A. terreus, A. fumigatus, A. versicolor* | Treatment with **posaconazole** (5 mg/kg via oral q12h) after one month after negative urine culture. In case of relapse, treatment was re-initiated. Clinical remission, but relapsed after treatment was discontinued. Average treatment time for all dogs was 7.25 months (range 1.5–15 months). Mean survival time for 9 of the 10 dogs was 241 days (range 44–516 days). | Remission and later relapse. |
| Corrigan *et al*., 2016 | USA | Disseminated aspergillosis | *A. terreus, A. fumigatus, A. versicolor* | Treatment with **posaconazole** (5 mg/kg via oral q12h) after one month after negative urine culture. In case of relapse, treatment was re-initiated. Clinical remission, but relapsed after treatment was discontinued. Average treatment time for all dogs was 7.25 months (range 1.5–15 months). Mean survival time for 9 of the 10 dogs was 241 days (range 44–516 days). | Remission and later relapse. |
| Corrigan *et al*., 2016 | USA | Disseminated aspergillosis | *A. terreus, A. fumigatus, A. versicolor* | Treatment with **posaconazole** (5 mg/kg via oral q12h) after one month after negative urine culture. In case of relapse, treatment was re-initiated. Clinical remission but then lost to follow-up during treatment. Average treatment time for all dogs was 7.25 months (range 1.5–15 months). Mean survival time for 9 of the 10 dogs was 241 days (range 44–516 days) | Remission, but no later outcome reported. |
| Corrigan *et al*., 2016 | USA | Disseminated aspergillosis | *A. terreus, A. fumigatus, A. versicolor* | Treatment with **posaconazole** (5 mg/kg via oral q12h) after one month after negative urine culture. In case of relapse, treatment was re-initiated. Clinical remission but then lost to follow-up during treatment. Clinically improved, relapsed and later died or were euthanized. Average treatment time for all dogs was 7.25 months (range 1.5–15 months). Mean survival time for 9 of the 10 dogs was 241 days (range 44–516 days) | Clinical improvement but later relapse and no recovery. |
| Corrigan *et al*., 2016 | USA | Disseminated aspergillosis | *A. terreus, A. fumigatus, A. versicolor* | Treatment with **posaconazole** (5 mg/kg via oral q12h) after one month after negative urine culture. In case of relapse, treatment was re-initiated. Clinical remission but then lost to follow-up during treatment. Clinically improved, relapsed and later died or were euthanized. Average treatment time for all dogs was 7.25 months (range 1.5–15 months). Mean survival time for 9 of the 10 dogs was 241 days (range 44–516 days) | Clinical improvement but later relapse and no recovery. |
| Corrigan *et al*., 2016 | USA | Disseminated aspergillosis | *A. terreus, A. fumigatus, A. versicolor* | Treatment with **posaconazole** (5 mg/kg via oral q12h) after one month after negative urine culture. In case of relapse, treatment was re-initiated. Clinical remission but then lost to follow-up during treatment. Clinically improved, relapsed and later died or were euthanized. Average treatment time for all dogs was 7.25 months (range 1.5–15 months). Mean survival time for 9 of the 10 dogs was 241 days (range 44–516 days) | Clinical improvement but later relapse and no recovery. |
| Corrigan *et al*., 2016 | USA | Disseminated aspergillosis | *A. terreus, A. fumigatus, A. versicolor* | Treatment with **posaconazole** (5 mg/kg via oral q12h) after one month after negative urine culture. In case of relapse, treatment was re-initiated. Clinical remission but then lost to follow-up during treatment. Clinically improved, relapsed and later died or were euthanized. Average treatment time for all dogs was 7.25 months (range 1.5–15 months). Mean survival time for 9 of the 10 dogs was 241 days (range 44–516 days) | Clinical improvement but later relapse and no recovery. |
| Corrigan *et al*., 2016 | USA | Disseminated aspergillosis | *A. terreus, A. fumigatus, A. versicolor* | Treatment with **posaconazole** (5 mg/kg via oral q12h) after one month after negative urine culture. In case of relapse, treatment was re-initiated. Clinical remission but then lost to follow-up during treatment. Clinically improved, relapsed and later died or were euthanized. Average treatment time for all dogs was 7.25 months (range 1.5–15 months). Mean survival time for 9 of the 10 dogs was 241 days (range 44–516 days). | Clinical improvement but later relapse and no recovery. |
| Corrigan *et al*., 2016 | USA | Disseminated aspergillosis | *A. terreus, A. fumigatus, A. versicolor* | Treatment with **posaconazole** (5 mg/kg via oral q12h) after one month after negative urine culture. In case of relapse, treatment was re-initiated. Clinical remission but then lost to follow-up during treatment. Clinically improved, relapsed and later died or were euthanized Average treatment time for all dogs was 7.25 months (range 1.5–15 months). Mean survival time for 9 of the 10 dogs was 241 days (range 44–516 days). | Clinical improvement but later relapse and no recovery. |
| Corrigan *et al*., 2016 | USA | Disseminated aspergillosis | *A. terreus, A. fumigatus, A. versicolor* | Treatment with **posaconazole** (5 mg/kg via oral q12h) after one month after negative urine culture. In case of relapse, treatment was re-initiated. Clinically improved an no relapse. Average treatment time for all dogs was 7.25 months (range 1.5–15 months). Mean survival time for 9 of the 10 dogs was 241 days (range 44–516 days). | Apparent recovery. |
| Magno *et al*., 2022 | Italy | Disseminated aspergillosis (iliac wing) | *A. terreus* | Moderate sensitivity to itraconazole (later decreased), resistance to fluconazole and a good sensitivity to voriconazole**.** Treatment with **itraconazole** (10 mg/kg q24h via oral and later increased to 7 mg/kg q12h) and intravenous fluid therapy preferred by the owner due to financial constraints. One year after initial treatment, treatment was discontinued due to vomiting and two weeks later the dog was euthanized due to worsening of the lameness. | No remission after long treatment. |
| Taylor *et al*., 2015 | USA | Central neurvous system aspergillosis | *A. terreus* | Treatment with **voriconazole** (5 mg/kg via oral q12 h) and terbinafine and amphotericin B. The dog was ambulatory tetraparetic with a head tilt to the right, after dedicated, rigorous physical rehabilitation and 11 months of antifungal treatment. | No remission after long treatment. |
| Taylor *et al*., 2015 | USA | Central neurvous system aspergillosis | *A. terreus* | Treatment with **fluconazole** (5 mg/kg IV q12 h) for 9 days, and discharged with **voriconzaole** (4.9 mg/kg via oral q12 h) and terbinafine. The dog was lost to follow-up after discharge. | Lost to follow-up. |
| Kano *et al.,* 2019 | Japan | Disseminated aspergillosis (iliac lymph node) | *A. caninus* | Treatment with **fluconazole** was initiated (4 mg/kg via oral twice a day) for 14 days. The isolate showed susceptibility to itraconazole and therefore therapy was replaced by **itraconazole** (8 mg/kg once a day) for 209 days. Due to lack of improvement and new susceptibility results, a **voriconazole** treatment was initiated (3.7 mg/kg via oral twince a day) but caused side effects. Therefore, therapy was again replaced by **itraconazole** with a (8 mg/kg, once a day). | No final outcome, difficult to assess effect. |
| Yang *et al*., 2020 | Australia | Disseminated aspergillosis (cervical lymph nodes) | *A. caninus* | Apart from antibiotic and painkillers for the coughing, the dog was treated with **itraconazole** (7.5 mg/kg via oral every 12 hours) indefinitely together with S-adenosylmethionine (to control hepatopathy). No apparent adverse effects from the medications were observed and the coughing improved although an sporadic dry cough was often observed. The lesions due to fungal infection remain stable but did not completely improved or resolved. Additional antifungal treatment was refused by the owner, although initial treatment with itraconazole was still continued. | Stable but no remission. |
| Pilkington *et al*., 2022 | England | Sinonasal mycosis | *A. fumigatus* | During frontal sinusotomy, the sinuses were flushed with a warm saline (0.9% NaCl) solution and **clotrimazole** (200 mg of 1% solution). Prior to closure of the overlying soft tissues, 400 mg of **clotrimazole** 1% cream was deposited in the frontal sinus. Discharge resolved 118 days later, but the dog was euthanized due to status epilecticus | Apparent remission together with sinusotomy, but dead due to other reasons. |
| Pilkington *et al*., 2022 | England | Sinonasal mycosis | *A. fumigatus* | Frontal sinusotomy and **clotrimazole** (200 mg of 1% solution) was administered directly to the sinus via an inwelling catheter once a day for 4 days. On day 5, **clotrimazole** 1% cream (200 mg) was deposited in the sinus, and the catheter was removed. Nasal discharged improved, but relapsed. Two more sinusotomies were performed with additional clotrimazole treatment. About 481 days later, intermittent mucopurulent nasal discharge was still present. | No remission. |
| Taylor *et al*., 2015 | USA | Central nervous system aspergillosis | *A. fumigatus* | Treatment with **fluconazole** (3.3 mg/kg via oral q12 h), terbinafine and levetiracetam (33 mg/kg PO q12 h). Chemotherapy was discontinued and the prednisone dosage was decreased. One month after diagnosis the dog was admitted to an emergency clinic because of status epilepticus and was euthanized | No remission. |
| Ygreda, Andrade, and Jara, 2021 | Peru | Fungal otitis | *A. niger* | Treatment with **itraconazole** (5mg/kg) and silimarina (500 mg) both via oral every 24 horas during 30 days. In addition, topical **ketoconazole** (2 g) together with antibiotic and corticoids on the affected ear for 45 days. 60 days of inconsistent treatment, *Aspergillus* was not found on culture, but clinical signs on the ears remained similar and therefore antibiotic and corticoids were prolonged together with an ear cleaning routine. The dog recovered. | Apparent recovery. |
