# Supplementary material for: Azole resistance in Aspergillus isolates from animals or their direct environment (2013–2023): a systematic review
Source: Front Vet Sci. 2025 Mar 20;12:1507997. doi: 10.3389/fvets.2025.1507997 (PMC11967370; doi:10.3389/fvets.2025.1507997)
Supplement: Supplementary file 3 [file Table_3.docx]

Supplementary Table 3: Summary of studies on assessing the presence of azole resistant aspergilli among *Aspergillus* isolates from animals or their environment or in environmental samples – results of studies using azole-containing agar plates.^[[1]](#footnote-1)^

^a^ Psittaciformes, Falconiformes, Passeriformes, Sphenisciformes, Accipitriformes, Columbiformes, Galliformes, Gruiformes, Anseriformes, Strigiformes, Charadriiformes, Ciconiiformes, Pelecaniformes, Trogoniformes, Bucerotiformes, Otidiformes; ^b^ Penguins, owls, Andalusian hens, Koala, Ring-tailed lemur, dolphin, orangutan, chimpanzee, psittacines, turacos, birds of prey, flamingos, mandrill, babonm, gorilla, pelicans, otter, rhea, emu, vulture;

^c^ for 5 of the 10 isolates the decreased susceptibility to itraconazole subsequently was confirmed by broth microdilution (NOTE: according to Table 2 in the respective paper 10 samples yielded growth on the itraconazole containing agar, while the text mentions growth for nine samples);

^d^ for 4 of the 7 isolates the decreased susceptibility to voriconazole was confirmed by broth microdilution;

^e^ NT, not tested.

1. (Viegas *et al.*, 2018; Sabino *et al.*, 2019; Barber *et al.*, 2020; Melo *et al.*, 2021; Álvarez-Pérez *et al.*, 2023; Bralet *et al.*, 2024) [↑](#footnote-ref-1)
