# Supplementary material for: Azole resistance in Aspergillus isolates from animals or their direct environment (2013–2023): a systematic review
Source: Front Vet Sci. 2025 Mar 20;12:1507997. doi: 10.3389/fvets.2025.1507997 (PMC11967370; doi:10.3389/fvets.2025.1507997)
Supplement: Supplementary file 4 [file Table_4.docx]

Supplementary Table 4: Summary of studies on the *in-vitro* activity of azoles on *Aspergillus fumigatus* isolates from animals or their environment – results of studies using method based on broth (micro)dilution and gradient diffusion, both yielding MIC values (µg/mL).^[[1]](#footnote-1)^

The red boxes highlight MIC parameters higher than the ECOFF. BMT, broth microdilution; GDT, gradient diffusion; NI, no/not enough information; NM, not mentioned. ^a^ Chicken, fowl, ducks, human, environment; ^b^ Chicken (N=25), geese (N=17), turkeys (N=13), ducks (N=5); the 20 isolates were included in the 60, but more concentrations of itraconazole were tested and more azoles; ^c^ Psittaciformes, Falconiformes, Passeriformes, phenisciformes, Accipitriformes, Columbiformes, Galliformes, Gruiformes, Anseriformes, Strigiformes, Charadriiformes, Ciconiiformes, Pelecaniformes, Trogoniformes, Bucerotiformes, Otidiformes; ^d^ Parrot, freshwater bird, sea birds, raptors; ^e^ Penguins, owls, Andalusian hens, Koala, Ring-tailed lemur, dolphin, orangutan, chimpanzee, psittacines, turacos, birds of prey, flamingos, mandrill, babonm, gorilla, pelicans, otter, rhea, emu, vulture; ^f^ Assuming the Y-axis of Figure 1 is the number of isolates instead of the percentage of isolates; ^g^ 95%-Interquartile: 0.125–0.5 µg/mL; ^h^ 95%-Interquartile: 0.015-0.125 µg/mL; ^i^ 95%-Interquartile: 0.031–0.25 µg/mL.

Supplementary Table 4: Summary of studies on the *in-vitro* activity of azoles on *Aspergillus fumigatus* isolates from animals or their environment – results of studies using method based on broth (micro)dilution and gradient diffusion, both yielding MIC values (µg/mL) – continued.^7^

The red boxes highlight MIC parameters higher than the ECOFF.

BMT, broth microdilution; GDT, gradient diffusion; NI, no/not enough information; NM, not mentioned.

^a^ Chicken, fowl, ducks, human, environment; ^b^ Chicken (N=25), geese (N=17), turkeys (N=13), ducks (N=5); the 20 isolates were included in the 60, but more concentrations of itraconazole were tested and more azoles; ^c^ Psittaciformes, Falconiformes, Passeriformes, Sphenisciformes, Accipitriformes, Columbiformes, Galliformes, Gruiformes, Anseriformes, Strigiformes, Charadriiformes, Ciconiiformes, Pelecaniformes, Trogoniformes, Bucerotiformes, Otidiformes; ^d^ Parrot, freshwater bird, sea birds, raptors; ^e^ Penguins, owls, Andalusian hens, Koala, Ring-tailed lemur, dolphin, orangutan, chimpanzee, psittacines, turacos, birds of prey, flamingos, mandrill, babonm, gorilla, pelicans, otter, rhea, emu, vulture; ^f^ Assuming the Y-axis of Figure 1 is the number of isolates instead of the percentage of isolates; ^g^ 95%-Interquartile: 0.125–0.5 µg/mL; ^h^ 95%-Interquartile: 0.015-0.125 µg/mL; ^i^ 95%-Interquartile: 0.031–0.25 µg/mL.

**Supplementary Table 4: Summary of studies on the *in-vitro* activity of azoles on *Aspergillus fumigatus* isolates from animals or their environment – results of studies using method based on broth (micro)dilution and gradient diffusion, both yielding MIC values (µg/mL) – continued.^7^**

The red boxes highlight MIC parameters higher than the ECOFF.

BMT, broth microdilution; GDT, gradient diffusion; NI, no/not enough information; NM, not mentioned.

^a^ Chicken, fowl, ducks, human, environment; ^b^ Chicken (N=25), geese (N=17), turkeys (N=13), ducks (N=5); the 20 isolates were included in the 60, but more concentrations of itraconazole were tested and more azoles; ^c^ Psittaciformes, Falconiformes, Passeriformes, Sphenisciformes, Accipitriformes, Columbiformes, Galliformes, Gruiformes, Anseriformes, Strigiformes, Charadriiformes, Ciconiiformes, Pelecaniformes, Trogoniformes, Bucerotiformes, Otidiformes; ^d^ Parrot, freshwater bird, sea birds, raptors; ^e^ Penguins, owls, Andalusian hens, Koala, Ring-tailed lemur, dolphin, orangutan, chimpanzee, psittacines, turacos, birds of prey, flamingos, mandrill, babonm, gorilla, pelicans, otter, rhea, emu, vulture; ^f^ Assuming the Y-axis of Figure 1 is the number of isolates instead of the percentage of isolates; ^g^ 95%-Interquartile: 0.125–0.5 µg/mL; ^h^ 95%-Interquartile: 0.015-0.125 µg/mL; ^i^ 95%-Interquartile: 0.031–0.25 µg/mL.

Supplementary Table 4: Summary of studies on the *in-vitro* activity of azoles on *Aspergillus fumigatus* isolates from animals or their environment – results of studies using method based on broth (micro)dilution and gradient diffusion, both yielding MIC values (µg/mL) – continued.^7^

The red boxes highlight MIC parameters higher than the ECOFF.

BMT, broth microdilution; GDT, gradient diffusion; NI, no/not enough information; NM, not mentioned.

^a^ Chicken, fowl, ducks, human, environment; ^b^ Chicken (N=25), geese (N=17), turkeys (N=13), ducks (N=5); the 20 isolates were included in the 60, but more concentrations of itraconazole were tested and more azoles; ^c^ Psittaciformes, Falconiformes, Passeriformes, Sphenisciformes, Accipitriformes, Columbiformes, Galliformes, Gruiformes, Anseriformes, Strigiformes, Charadriiformes, Ciconiiformes, Pelecaniformes, Trogoniformes, Bucerotiformes, Otidiformes; ^d^ Parrot, freshwater bird, sea birds, raptors; ^e^ Penguins, owls, Andalusian hens, Koala, Ring-tailed lemur, dolphin, orangutan, chimpanzee, psittacines, turacos, birds of prey, flamingos, mandrill, babonm, gorilla, pelicans, otter, rhea, emu, vulture; ^f^ Assuming the Y-axis of Figure 1 is the number of isolates instead of the percentage of isolates; ^g^ 95%-Interquartile: 0.125–0.5 µg/mL; ^h^ 95%-Interquartile: 0.015-0.125 µg/mL; ^i^ 95%-Interquartile: 0.031–0.25 µg/mL.

Supplementary Table 4: Summary of studies on the *in-vitro* activity of azoles on *Aspergillus fumigatus* isolates from animals or their environment – results of studies using method based on broth (micro)dilution and gradient diffusion, both yielding MIC values (µg/mL) – continued.^7^

The red boxes highlight MIC parameters higher than the ECOFF.

BMT, broth microdilution; GDT, gradient diffusion; NI, no/not enough information; NM, not mentioned.

^a^ Chicken, fowl, ducks, human, environment; ^b^ Chicken (N=25), geese (N=17), turkeys (N=13), ducks (N=5); the 20 isolates were included in the 60, but more concentrations of itraconazole were tested and more azoles; ^c^ Psittaciformes, Falconiformes, Passeriformes, Sphenisciformes, Accipitriformes, Columbiformes, Galliformes, Gruiformes, Anseriformes, Strigiformes, Charadriiformes, Ciconiiformes, Pelecaniformes, Trogoniformes, Bucerotiformes, Otidiformes; ^d^ Parrot, freshwater bird, sea birds, raptors; ^e^ Penguins, owls, Andalusian hens, Koala, Ring-tailed lemur, dolphin, orangutan, chimpanzee, psittacines, turacos, birds of prey, flamingos, mandrill, babonm, gorilla, pelicans, otter, rhea, emu, vulture; ^f^ Assuming the Y-axis of Figure 1 is the number of isolates instead of the percentage of isolates; ^g^ 95%-Interquartile: 0.125–0.5 µg/mL; ^h^ 95%-Interquartile: 0.015-0.125 µg/mL; ^i^ 95%-Interquartile: 0.031–0.25 µg/mL.

Supplementary Table 4: Summary of studies on the *in-vitro* activity of azoles on *Aspergillus fumigatus* isolates from animals or their environment – results of studies using method based on broth (micro)dilution and gradient diffusion, both yielding MIC values (µg/mL) – continued.^7^

The red boxes highlight MIC parameters higher than the ECOFF.

BMT, broth microdilution; GDT, gradient diffusion; NI, no/not enough information; NM, not mentioned.

^a^ Chicken, fowl, ducks, human, environment; ^b^ Chicken (N=25), geese (N=17), turkeys (N=13), ducks (N=5); the 20 isolates were included in the 60, but more concentrations of itraconazole were tested and more azoles; ^c^ Psittaciformes, Falconiformes, Passeriformes, Sphenisciformes, Accipitriformes, Columbiformes, Galliformes, Gruiformes, Anseriformes, Strigiformes, Charadriiformes, Ciconiiformes, Pelecaniformes, Trogoniformes, Bucerotiformes, Otidiformes; ^d^ Parrot, freshwater bird, sea birds, raptors; ^e^ Penguins, owls, Andalusian hens, Koala, Ring-tailed lemur, dolphin, orangutan, chimpanzee, psittacines, turacos, birds of prey, flamingos, mandrill, babonm, gorilla, pelicans, otter, rhea, emu, vulture; ^f^ Assuming the Y-axis of Figure 1 is the number of isolates instead of the percentage of isolates; ^g^ 95%-Interquartile: 0.125–0.5 µg/mL; ^h^ 95%-Interquartile: 0.015-0.125 µg/mL; ^i^ 95%-Interquartile: 0.031–0.25 µg/mL.

1. (Voelter-Ratson *et al.*, 2014; Wang *et al.*, 2014; Ziółkowska, Tokarzewski and Nowakiewicz, 2014; Talbot *et al.*, 2015; Bunskoek *et al.*, 2017; Tartor and Hassan, 2017; Sarrafha *et al.*, 2018; Valdes *et al.*, 2018; Cullen *et al.*, 2019; Nawrot *et al.*, 2019; Vedova *et al.*, 2019; Ohno *et al.*, 2019; Barber *et al.*, 2020; Mustikka, Grönthal and Pietilä, 2020; Roberts *et al.*, 2020; Spanamberg *et al.*, 2020; Melo *et al.*, 2021; Brito Devoto *et al.*, 2022; Cateau *et al.*, 2022; Cruciani *et al.,* 2022; Lofgren *et al.*, 2022; Martinez *et al.*, 2022; Thomson *et al.*, 2022; Mutlu Sariguzel *et al.*, 2023; Álvarez-Pérez *et al.*, 2023; Bendary *et al.*, 2023; Debergh *et al.*, 2023; Bralet *et al.*, 2024; Uchida-Fujii *et al.*, 2024) [↑](#footnote-ref-1)
