# Supplementary material for: Azole resistance in Aspergillus isolates from animals or their direct environment (2013–2023): a systematic review
Source: Front Vet Sci. 2025 Mar 20;12:1507997. doi: 10.3389/fvets.2025.1507997 (PMC11967370; doi:10.3389/fvets.2025.1507997)
Supplement: Supplementary file 5 [file Table_5.docx]

Supplementary Table 5: Summary of studies on the *in-vitro* activity of azoles on *Aspergillus flavus* isolates from animals or their environment – results of studies using method based on broth (micro)dilution and gradient diffusion, both yielding MIC values (µg/mL).^[[1]](#footnote-1)^

The red boxes highlight MIC parameters higher than the ECOFF.

BMT, broth microdilution; GDT, gradient diffusion; NI, no/not enough information; NM, not mentioned.

^a^ Chicken, fowl, ducks, human, environment.

Supplementary Table 5: Summary of studies on the *in-vitro* activity of azoles on *Aspergillus flavus* isolates from animals or their environment – results of studies using method based on broth (micro)dilution and gradient diffusion, both yielding MIC values (µg/mL) – continued.^8^

The red boxes highlight MIC parameters higher than the ECOFF.

BMT, broth microdilution; GDT, gradient diffusion; NI, no/not enough information; NM, not mentioned.

^a^ Chicken, fowl, ducks, human, environment.

1. (Sarrafha *et al.*, 2018; Cullen *et al.*, 2019; Vedova *et al.*, 2019; Mustikka, Grönthal and Pietilä, 2020; Roberts *et al.*, 2020; Kano *et al.*, 2021; Brito Devoto *et al.*, 2022; Cruciani *et al.*, 2022; Mutlu Sariguzel *et al.*, 2023; Uchida-Fujii *et al.*, 2024) [↑](#footnote-ref-1)
