# Supplementary material for: Azole resistance in Aspergillus isolates from animals or their direct environment (2013–2023): a systematic review
Source: Front Vet Sci. 2025 Mar 20;12:1507997. doi: 10.3389/fvets.2025.1507997 (PMC11967370; doi:10.3389/fvets.2025.1507997)
Supplement: Supplementary file 6 [file Table_6.docx]

Supplementary Table 6: Summary of studies on the *in-vitro* activity of azoles on other *Aspergillus* species than *A. fumigatus* and *A. flavus*, and on isolates not further identified than *Aspergillus* genus or a specific section, from animals or their environment – results of studies using method based on broth (micro)dilution and gradient diffusion, both yielding MIC values (µg/mL).^[[1]](#footnote-1)^

For *Aspergillus* species in printed in green (T)ECOFF are available and the number of isolates with a MIC value higher than the ECOFF value is reported, if applicable; DD, disk diffusion; MD, microdilution; BMT, broth microdilution; GDT, gradient diffusion; NI, no/not enough information; NM, not mentioned.^a^ Chicken, fowl, ducks, human, environment; ^b^ Humans, squirrels, air laying hen farms; ^c^ Penguins, owls, Andalusian hens, Koala, Ring-tailed lemur, dolphin, orangutan, chimpanzee, psittacines, turacos, birds of prey, flamingos, mandrill, babonm, gorilla, pelicans, otter, rhea, emu, vulture.

Supplementary Table 6: Summary of studies on the *in-vitro* activity of azoles on other *Aspergillus* species than *A. fumigatus* and *A. flavus*, and on isolates not further identified than *Aspergillus* genus or a specific section, from animals or their environment – results of studies using method based on broth (micro)dilution and gradient diffusion, both yielding MIC values (µg/mL) - continued.^9^

For *Aspergillus* species in printed in green (T)ECOFF are available and the number of isolates with a MIC value higher than the ECOFF value is reported, if applicable;

DD, disk diffusion; MD, microdilution; BMT, broth microdilution; GDT, gradient diffusion; NI, no/not enough information; NM, not mentioned.

^a^ Chicken, fowl, ducks, human, environment; ^b^ Humans, squirrels, air laying hen farms; ^c^ Penguins, owls, Andalusian hens, Koala, Ring-tailed lemur, dolphin, orangutan, chimpanzee, psittacines, turacos, birds of prey, flamingos, mandrill, babonm, gorilla, pelicans, otter, rhea, emu, vulture.

Supplementary Table 6: Summary of studies on the *in-vitro* activity of azoles on other *Aspergillus* species than *A. fumigatus* and *A. flavus*, and on isolates not further identified than *Aspergillus* genus or a specific section, from animals or their environment – results of studies using method based on broth (micro)dilution and gradient diffusion, both yielding MIC values (µg/mL) - continued.^9^

For *Aspergillus* species in printed in green (T)ECOFF are available and the number of isolates with a MIC value higher than the ECOFF value is reported, if applicable;

DD, disk diffusion; MD, microdilution; BMT, broth microdilution; GDT, gradient diffusion; NI, no/not enough information; NM, not mentioned.

^a^ Chicken, fowl, ducks, human, environment; ^b^ Humans, squirrels, air laying hen farms; ^c^ Penguins, owls, Andalusian hens, Koala, Ring-tailed lemur, dolphin, orangutan, chimpanzee, psittacines, turacos, birds of prey, flamingos, mandrill, babonm, gorilla, pelicans, otter, rhea, emu, vulture.

Supplementary Table 6: Summary of studies on the *in-vitro* activity of azoles on other *Aspergillus* species than *A. fumigatus* and *A. flavus*, and on isolates not further identified than *Aspergillus* genus or a specific section, from animals or their environment – results of studies using method based on broth (micro)dilution and gradient diffusion, both yielding MIC values (µg/mL) - continued.^9^

For *Aspergillus* species in printed in green (T)ECOFF are available and the number of isolates with a MIC value higher than the ECOFF value is reported, if applicable;

DD, disk diffusion; MD, microdilution; BMT, broth microdilution; GDT, gradient diffusion; NI, no/not enough information; NM, not mentioned.

^a^ Chicken, fowl, ducks, human, environment; ^b^ Humans, squirrels, air laying hen farms; ^c^ Penguins, owls, Andalusian hens, Koala, Ring-tailed lemur, dolphin, orangutan, chimpanzee, psittacines, turacos, birds of prey, flamingos, mandrill, babonm, gorilla, pelicans, otter, rhea, emu, vulture.

1. (Kano *et al*., 2013; Voelter-Ratson *et al.*, 2014; Wang *et al.*, 2014; Ziółkowska, Tokarzewski and Nowakiewicz, 2014; Kano *et al*., 2015; Talbot *et al.*, 2015; Lim *et al*., 2016; Sabino *et al*., 2016; Ayandiran and Dahunsi, 2017; Tartor and Hassan, 2017; Sarrafha *et al.*, 2018; Valdes *et al.*, 2018; Cullen *et al.*, 2019; Kano *et al*., 2019; Nawrot *et al.*, 2019; Barrs *et al*., 2013; Vedova and Della, 2019; Mustikka, Grönthal and Pietilä, 2020; Reed *et al*., 2020; Roberts *et al.*, 2020; Spanamberg *et al.*, 2020; Yang *et al*., 2020; Kay *et al*., 2021; Meade *et al*., 2021; Rashid et al., 2021; Cruciani *et al.*, 2022; Martinez *et al.*, 2022; Thomson *et al.*, 2022; Mo, Picard and Gummow, 2023; Mutlu Sariguzel *et al.*, 2023; Bendary *et al.*, 2023; Uchida-Fujii *et al.*, 2024) [↑](#footnote-ref-1)
