# Supplementary material for: Azole resistance in Aspergillus isolates from animals or their direct environment (2013–2023): a systematic review
Source: Front Vet Sci. 2025 Mar 20;12:1507997. doi: 10.3389/fvets.2025.1507997 (PMC11967370; doi:10.3389/fvets.2025.1507997)
Supplement: Supplementary file 7 [file Table_7.docx]

Supplementary Table 7: Summary of studies on the *in-vitro* activity of azoles on *Aspergillus* isolates from animals or their environment – results of studies using a disk diffusion method.^[[1]](#footnote-1)^

NM, not mentioned; GM, geometric mean; R, resistant, I, intermediate susceptible, and S, susceptible (according to the interpretation by the authors).

Supplementary Table 7: Summary of studies on the *in-vitro* activity of azoles on *Aspergillus* isolates from animals or their environment – results of studies using a disk diffusion method - continued.^10^

NM, not mentioned; GM, geometric mean; R, resistant, I, intermediate susceptible, and S, susceptible (according to the interpretation by the authors).

1. (Ziółkowska, Tokarzewski and Nowakiewicz, 2014; Afshan Naz and Shafique, 2017; Tartor and Hassan, 2017; Aneke *et al.*, 2018; Viegas *et al.*, 2018; Hampson *et al.*, 2019; Sabino *et al.*, 2019b; Sadiek, El and Amen, 2019; Barber *et al.*, 2020; Fahmy, Mahrous and Sayed-Elahl, 2020; Tawab *et al.*, 2020; Melo *et al.*, 2021; Mgbeahuruike *et al.*, 2021; Mohammed and Abdel-Latef, 2021; Ygreda, Andrade and Jara, 2021; Ralte *et al.*, 2022; Álvarez-Pérez *et al.*, 2023; El-Hamaky *et al.*, 2023; Colombo *et al.*, 2024) [↑](#footnote-ref-1)
